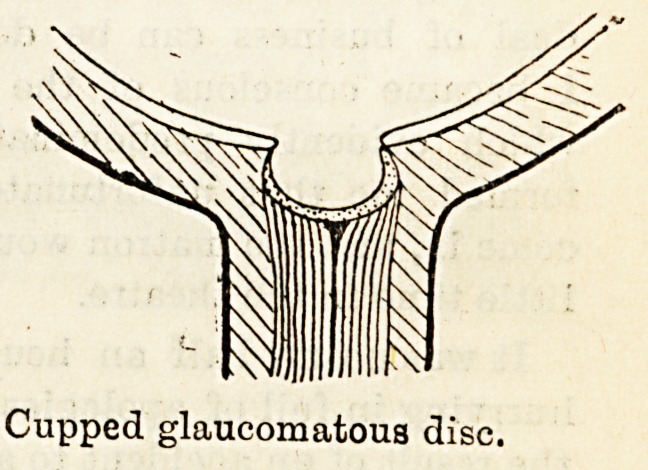# The Hospital. Nursing Section

**Published:** 1903-09-19

**Authors:** 


### The Hospital.

**Nursing Section.**

Contributions for this Section of "The Hospital" should be addressed to the Editor, "The Hospital" Nursing Section, 28 & 29 Southampton Street, Strand, London, W.C.

No. 886.—Vol. XXXIV. *SATURDAY, SEPTEMBER* 19, 1903.

### Notes on News from the Nursing World.

### OUR CHRISTMAS DISTRIBUTION.

Our readers will understand that our only desire in reminding them again of our Christmas distribution of articles of clothing for the use of patients in hospitals and infirmaries, is because we know that in order to maintain interest in any matter it is advisable to make frequent reference to it. Moreover, we should like to be able to announce, in our first number for October, that the first parcel has arrived, and from that date onward to chronicle each week the receipt of welcome contributions. These should in all cases be addressed to the Editor, 28 & 29 Southampton Street, Strand, London, W.C., with "Clothing Distribution" inscribed on the outside of the parcel.

### THE MATRONSHIP OF THE CANCER HOSPITAL.

The Committee of the Cancer Hospital, Fulham Road, are about to choose a new matron, in the room of Miss Rogers. The salary is £80 per annum, and candidates must be between 30 and 45 years of age. Under the auspices of Miss Rogers the Nurses' Home which was specially built for the use of the night staff, was opened in the early part of 1901. This, however, is only one of many improvements which took place during her long term of office. She was appointed assistant matron in 1884, and in 1885 was promoted to the post of matron. The lectures which are given in elementary nursing at the Cancer Hospital by the sister in charge of the theatre were originated by her, and she spared no pains to make the diet of the nurses liberal and varied.

### ROYAL CORNWALL INFIRMARY.

It is pleasant to learn from the account of our Commissioner's visit to the Royal Cornwall Infirmary, at Truro, this week, that every effort is being made in the most western and the most youthful, of cities to come into line with the modern system of nursing. Not only is the period of training three years, but the teaching is of a thoroughly practical and up to date character. The recently appointed matron, who has enjoyed experience at several important institutions, seems to have entered with spirit into her work, and to have already effected improvements in the nurses' quarters. It may be hoped that in time, when the infirmary is enlarged, she will be able to secure for the day nurses separate bedrooms of their own instead of cubicles, which, however spacious, are objectionable.

### PROPOSED BONUS FOR POOR LAW NURSES.

The Hull Board of Guardians, having experienced great difficulty in keeping nurses in the workhouse infirmary, have decided to resort to the expedient of trying to tempt them to stay by offering them a bonus of £5 per annum, provided that they remain in the service of the Board for a period of three years. It will be interesting to see whether the Local Government Board will confirm the action of the guardians. Our own view is that the best way for boards of guardians to keep nurses is to pay reasonable salaries, to study their comfort and convenience in respect to accommodation, diet, duty, and holidays, rather than by means of a reward in money which can only be secured at the end of three years.

### CHANGES AT KING'S LYNN HOSPITAL.

Complaints reach us that there is not a private nurse fully trained to be had from the West Norfolk and King's Lynn Hospital, and that changes in the staff have been so frequent lately that nearly a score of nurses, old and new, have left since last September. Bearing in mind the fact that the number of nurses employed at the hospital does not, according to the latest return, exceed a dozen, this is a startling statement; but it is made on the authority of an informant who should be in a position to know. If it be correct, the board would do well to institute inquiries as to the cause.

### THE COUNTESS OF SCARBROUGH AND THE PARTIALLY TRAINED NURSE.

At a garden party given in the grounds of Wadworth Hall, in aid of the funds of the Sandbeck Nursing Association, the Countess of Scarborough at the close of an interesting address, expressed a hope that the committee would not be discouraged if the scheme were not taken up keenly at first. The Sandbeck Nursing Association, it may be explained, was started in January, and is affiliated with the Holt Ockley Benefit Nursing Association. Possibly the difficulty in obtaining public support which Lady Scarborough seemed to think, might be encountered, if it has not already been experienced, would have been less formidable if the organisation had been formed on less debatable lines. It is quite true, as Lady Scarborough said, that the confidence of people cannot be secured in a moment. But in a nursing movement it is more likely to be obtained speedily when it is known that the nurses employed are fully qualified than when, in the supposed interests of economy, an association is content with those who are only partially trained.

### A SLANDER ON THE NURSING PROFESSION.

The master of Epsom Workhouse, who is honorary secretary of the National Association of Workhouse Masters and Matrons, is reported, in the course of an interview, to have said, "with a shrug," that he did not know "that there was more dependence to be placed upon nurses as a class than upon masters and matrons." He proceeded, according to the printed report, to observe that he did not think that "the masters and matrons would have come back in greater disgrace from South Africa than did the nurses." That this is a specimen of the fair-mindedness of the workhouse master we should be sorry to believe. The master at Epsom having presumably heard that a stray nurse or two in South Africa during the war did not behave satisfactorily, speaks of the whole body of ladies whose devoted attention to our sick and wounded soldiers in South Africa has been recognised by the Sovereign, as coming back in disgrace. The author of such a sweeping slander as this is not well qualified to discuss the relative merits or demerits of nurses as compared with those of workhouse masters and matrons.

### HOME AT BIRKENHEAD BOROUGH HOSPITAL.

On Saturday, the 26th instant, the new nurses' home built in connection with the Birkenhead Borough Hospital will be opened by Sir Elliott Lees, M.P. It is adjacent to the hospital, and the site was purchased a year or two ago from the Corporation of Birkenhead. The ground floor includes a dining-hall, a nurses' day-room, a head nurses' room, and nine bedrooms, access to these being obtained from a spacious and well-lighted corridor, at one end of which is placed the sanitary tower. At the back of the dining-hall are the administration offices. On the first floor are a general sitting-room, ten bedrooms for nurses, and accommodation for domestic servants. A feature is that the dining-hall and day-room can, if required, be converted into one room. The home is lighted by electricity.

### THE TESTIMONIAL QUESTION AT SOUTHAMPTON.

The Southampton Board of Guardians have determined, at the instance of the chairman, that testimonials or replies to questions as to character and capacity, are not to be given by the medical superintendent and the matron of the Incorporation Infirmary, unless they are authorised by the Board. This decision has been arrived at in consequence of a very awkward incident. A former nurse at Shirley Warren Infirmary had received a good testimonial from the board, but the matron, to whom application was made for references by a nursing institute, considered it her duty to answer certain questions put to her in a private letter, in a manner which had the effect of causing the nurse to lose the appointment. It is not suggested that in taking this course the matron was actuated by any feeling of ill-will, but as the guardians in their testimonial said that there was no complaint whatever to be made, and the matron in her letter expressed entirely different sentiments, there was quite sufficient justification for the action which the Southampton Guardians have since taken. A nurse has a right to expect that if she obtains a satisfactory testimonial from her board, no individual will be able to go behind it to her detriment.

### DISTRICT NURSING IN IRELAND.

At a conference held on Workhouse Reform in Ireland at Loughrea last week, one of the most interesting papers was read by Father O'Donovan on District Nursing. Defining a district nurse as a qualified person acting in a district which had a small town for a centre, going around bandaging, poulticing, helping, and, by her example, inclining to cleanliness in the house, Father O'Donovan said that she could also improve the cooking and render impossible what he had seen—a typhoid patient being fed on solid food, and a pan of milk for distribution standing in one corner of the sick-room. His practical recommendation was that an effort should be made to get a trained nurse for every district in Ireland, who should have a field of operations with a radius of three or four miles. He did not propose that she should be paid out of taxation; a hundred a year would, he thought, be enough for each district, and where a district was too poor to subscribe that amount resort might be had to the fund raised by Lady Dudley. Dr. Ryan and several other speakers concurred in the views of Father O'Donovan, and it is a legitimate conclusion that an earnest attempt will be made to secure for several centres in the sister island—where the sick poor need more attention than is provided by the Guardians—the blessing of a resident district nurse.

### THE TRAINED WORKHOUSE MATRON.

At the last meeting of the Berkhamsted Board of Guardians Mr. Cheald moved that the matron of the workhouse be appointed superintendent of the nursing department, and after some discussion the motion was carried. We do not think that the guardians could have pursued a wiser course. The matron of Berkhamsted Workhouse is a qualified and certificated nurse. She has always admittedly shown a keen desire to serve the best interests of the Union, and as her training is unimpeachable, there is no reason why she should not act both as matron and superintendent nurse.

### A SHAM NURSE AND A SHAM FORTUNE.

As we fully anticipated, the announcement made in a nursing paper, on the faith of a paragraph in one of the half-penny prints, that a person posing as a nurse had inherited a large fortune under romantic circumstances, turns out to be a fiction. The "nurse," instead of coming into an estate worth £3,000 a year, and £24,000 in money, has been sent to prison for 15 days for trying to defraud a draper in Paisley. We do not know whether the story that the woman "attended" a law student in Glasgow during an attack of small-pox is as false as that of her inheritance of a fortune. But it is as well that the bubble about a sham nurse and a sham fortune should have been pricked in a manner that does not admit of dispute.

### FOUR NURSES FOR A HUNDRED PATIENTS.

There is a member of the South Stoneham Board of Guardians, in Hampshire, who thinks that Mr. Baldwyn Fleming, one of the inspectors of the Local Government Board, who, with Dr. Fuller, has lately issued a report on the nursing in the workhouse, wants nurses to be provided with a palace like that of the Duke of Devonshire, "and servants to wait on the nurses." We do not know why servants should not wait on nurses who, in their turn, wait on patients. But the modest proposal of the inspectors that a nurses' home should be built in order to meet the urgent needs of the situation hardly lends itself to the satire of Mr. Hayward. At present there are generally over a hundred cases under treatment in the workhouse infirmary, and as the nursing staff is limited to a superintendent and three nurses, the inmates are, in spite of the regulations of the Local Government Board to the contrary, engaged to assist. This, it is obvious, should be remedied without delay, and as accommodation for an adequate number of nurses cannot be secured under existing conditions, we hope that the committee of the whole Board of Guardians, to whom the question has been referred, will decide to proceed at once with the erection of the suggested home.

### MINERS AND NURSING.

The Ashington Nursing Association seems to be popular with the miners of Northumberland. At the meeting of members, at which the annual report was submitted, the decision of the Ashington miners' and deputies' lodges to affiliate with the society was mentioned as an assurance that its work was appreciated in the district. The financial position is extremely satisfactory, the balance in hand at the end of the year being £116. The annual procession in aid of the fund realised £60, a result which also indicates the feeling of the class who derive the chief benefit from the labours of the two nurses working under the auspices of the association.

### A NEW ORGANISATION FOR GLASGOW.

Under the title of the "Glasgow General Nursing Association" an organisation has been formed in the City of Glasgow which has for its object the training of women to act as midwifery nurses to the middle and poorer classes. The promoters of the scheme contemplate the provision of efficient nurses for this specific purpose at reasonable charges, and it is proposed that there should be classes open to ladies who are not trained nurses. It is stated that it is in contemplation to extend the movement so as to include the training of nurses generally. But until that is done the title is misleading. Midwifery is not general nursing.

### ENTERTAINMENTS FOR NURSING ASSOCIATIONS.

Among the numerous efforts on behalf of nursing organisations at this period of the year, a fête at the Charterhouse grounds in aid of the Coventry Nursing Institution deserves mention. Happily, the weather was fine, and the beauty of the grounds attracted a large attendance. A small extra fee was paid by many of the visitors who availed themselves of the opportunity of inspecting a remarkable painting representing the Crucifixion which was discovered in 1899. At Romsey a carnival on behalf of the funds of the Royal South Hants and Southampton Hospital, and the Romsey Nursing Home, was quite the event of the season, thousands of visitors flocking into the town in order to see the decorations and illuminations. In the evening there was a torchlight procession, and it is understood that a substantial sum of money has been realised by the various entertainments.

### A CAUTION TO NURSES.

Last week we published a letter from a matron complaining that a cloak she sent on approbation to a nurse who answered her advertisement had not been paid for, the nurse having left her lodgings and taken the cloak with her. Another case has just been brought to our notice of a nurse replying to an advertiser who professed that she had a bicycle for which she had paid £10 10s., and was anxious to part with at a low figure. Being asked for details, the advertiser described herself as a nurse out of work, requiring ready money, who was unable to use the bicycle because she had been forbidden to ride owing to heart disease. Ultimately, the would-be purchaser transmitted £2 in order that she might have the bicycle on approval. She received a postcard acknowledging the receipt of the money, but no machine. Upon inquiry, the address given was found to be only a house for receiving letters, and the fact that seven more replies were awaiting the advertiser suggests that there had been more than one victim. Our correspondent does not state whether she put the matter into the hands of the police, the only thing to be done after the event. But prevention is better than cure, and we strongly advise nurses who send cash to advertisers of whom they know nothing to make use of the system of deferred money orders, of which they can obtain particulars at any Post Office. In such a case as that of our correspondent the £2 might have been saved if a deferred money order had been procured.

### A STREET UNIFORM IN CHICAGO.

Surprise and regret having been expressed by an Ohio nurse at seeing a great number of nurses in uniform at the stores, in the parks, and in church in Chicago, it has been explained that some of the trained nurses working in the city have a street uniform designed wholly for that purpose. This is obviously in order to avoid wearing the same uniform as that worn in the sick-room. In any case there seems to us to be no reason why the Ohio nurse should have been "shocked" at encountering nurses in uniform out of doors. It is, as we have said before, a great convenience for nurses who are off duty for a short time to be able to enjoy fresh air in uniform; and so long as they reflect no discredit upon it they should not be prohibited from the use of it outside the institution.

### AN EXCELLENT EXAMPLE.

A generous offer has been made by Mr. A. A. Adams, of Bennet's Castle House, Chadwell Heath, who has expressed his willingness to invest £1,000 in the names of two trustees, and to guarantee a minimum dividend of 5 per cent. per annum for the next five years, the dividend to be devoted towards the payment of trained nurses for Chadwell Heath, Beacontree Heath, and Dagenham. It is proposed that the nurses shall be under the direction of a small committee, and that the extra expenses shall be met by public subscription. We cannot doubt that, Mr. Adams having given such a stimulus to the undertaking, the committee and the public will do the rest.

### A NURSE AS MILK INSPECTOR.

It is stated that promoters of the pure-milk project, Milwaukee, United States, have practically decided to place a trained nurse in charge of the laboratory of the Milk Commission of the Children's Free Hospital, instead of a chemist.

### Lectures on Ophthalmic Nursing.

By A. S. COBBLEDICK, M.D., B.S.Lond., Senior Clinical Assistant and late House-Surgeon and Registrar to the Royal Eye Hospital.

### LECTURE XIX.—TREATMENT OF ACUTE GLAUCOMA (*continued*).—CHRONIC GLAUCOMA.

THE chief dangers of this operation are:—

1. Injury to the lens.
2. Expulsion of lens and vitreous due to great intra-ocular pressure.
3. Severe and continuous hæmorrhage resulting in loss of the eye.
4. Retching and vomiting after the operation as a result of the anæsthetic, causing the lens or vitreous to escape through the incision.

The after treatment of these operation cases often requires great vigilance on the part of the nurse to ensure a good result. If the patient has a distressing cough or he retches or vomits from the effect of the anæsthetic, it is apparent that there is considerable danger of expulsion of the lens and possibly vitreous, and, consequently, all sight in the eye may be lost. If any of these conditions are present a pad and bandage should be applied, and, in addition, a large pad of absorbent wool—as much as can be grasped in one hand—should be within reach of the patient; this must be firmly applied over the operation eye by the nurse when the patient retches or coughs. A similar precaution must be taken when the bowels act; in these acute cases there is not often time to give an aperient before the operation, but when possible a free evacuation of the bowels should be obtained before operation so that they will not act again for two or three days.

Troublesome patients—and they are not uncommon—have a tendency to remove their dressings, get out of bed, and surreptitiously introduce their septic fingers beneath the dressings: these tendencies are mentioned that they may be prevented, for many eyes have been lost through relaxing a vigilant watch—especially at night. In some cases it is well to prevent the possibility of the patient touching the dressing by limiting their range of hand movements by means of a bandage around each wrist attached to the foot or side of the bed.

The diet for the first two or three days following the operation should be light, but not necessarily fluid.

For 24 hours after operation the patient should lie flat on the back, with the head only slightly raised, and warned that the success of the operation largely depends on his keeping absolutely quiet in one position. For as long as four days after this operation the patient should move as little as possible; at the end of that time union of the wound has usually taken place, though this is by no means firm, and any strain on it will readily re-open it.

In irrigating the sac it is obvious that great care must be taken not to exert any pressure whatever on the eyeball.

When the patient is convalescent he should wear a protective spectacle or goggle, of which MacHardy's is a useful pattern.

*Chronic Glaucoma.*—As its name implies, the onset and progress of this disease is gradual and insidious. It is a serious condition, for in spite of all treatment most cases slowly but surely get worse, with increasing loss of vision, and finally blindness.

There are no acute symptoms to draw the patient's attention to the condition, and not unfrequently the disease is discovered more or less accidentally in the routine examination of the eyes.

*Symptoms.*—The chief complaint is of gradual loss of vision; the sight cannot be much improved by changing the glasses. Nearly all these cases suffer from hypermetropia, a condition in which the antero-posterior diameter of the eyeball is shorter than normal.

The field of vision diminishes, the scotoma beginning on the nasal side and gradually contracting towards the centre, from all sides.

*Signs.*—The external appearance of the eye is normal, the pupil reacts to light and accommodation, and there may be little to draw one's attention to this serious condition. Not unfrequently there is a difference in the *tension* of the two eyes, and this should at once arrest the attention and lead to a thorough examination. If there is a considerable rise of tension, the two pupils may be unequal in size and the anterior chamber *shallow* in the eye with the higher tension.

The ophthalmoscopic appearance of the disc must be the chief guide in making the diagnosis: the increased intra-ocular tension causes the disc to become *cupped.*

Normal disc. Cupped glaucomatous disc.

The accompanying diagrams show the difference between the normal disc and the glaucomatous disc. In viewing the disc with the ophthalmoscope the blood-vessels can be traced from the periphery of the retina to the edge of the disc, where they disappear from view: by using concave spheres, the blood-vessels again come into view on the floor of the cup, and in this manner the depth of the cup can be estimated.

This *complete* cupping must not be confused with physiological cupping, frequently found in normal eyes: this consists of a *gradual shelving* towards the centre of the disc, or it may be that the central portion only is cupped with no cupping of the outer half of the disc.

Sooner or later the cupped disc also becomes atrophic—a condition recognised by the whiteness of the disc and the smallness of the retinal arteries.

*Prognosis.*—This is never good: in spite of all treatment most cases get slowly but surely worse, and end in blindness. Occasionally the condition comes to a standstill.

The onset of white atrophy renders the case hopeless.

*Treatment.*—Near work must be forbidden, and correct glasses should be prescribed for distance. It is a question whether the use of a myotic, *e.g.* eserine sulphate or either an iridectomy or sclerotomy, is the best treatment; if an operation is performed the immediate result is some loss of vision, but it may retard the disease for a few years. Patients are often comfortable and satisfied with eserine gr. $\frac{1}{2}$ to i. ad. ℥i. of water, one or two drops three times a day; this serves to lower the tension and diminish discomfort.

If an operation is to be performed, the instruments and nursing are the same as in acute glaucoma, but it is well to have used eserine for 24 hours before the operation, and rendered the pupil almost a pin-point in size; by so doing there is less chance of the iris prolapsing after the section has been completed.

### Nursing at the Royal Cornwall Infirmary.

INTERVIEW WITH THE MATRON. BY OUR COMMISSIONER.

It had been raining hard for many hours as I drove up the steep hill which leads to the Royal Cornwall Infirmary in the cathedral city of Truro. But the downpour had ceased and the sun, which had suddenly emerged as if ashamed to have hidden his face so long, was being reflected alike in the wet stones and the glistening drops that decorated the leaves and the foliage around the hospital. The latter is a substantial-looking edifice overlooking the city, built of the dull-grey stone which harmonises so well with the scenery in the West of England, and standing on a piece of flat ground cut out from the side of the hill. The carriage drive after the big gates are passed is quite level, and so is a small portion behind the house, but everywhere else the gardens climb up and down the hill. The Prince of Wales's plumes appear on the facade, as on many public buildings in Cornwall. The matron's pleasant little room on the ground floor, is a happy combination of ease and work, its cosy cushions and pretty knick-knacks suggesting a drawing-room, whilst its solid-looking desk shows a good deal of business can be done there when required. As I became conscious of the delicate scent of honeysuckle which evidently predominated in the vases, a maid informed me that unfortunately an accident case had just come in, and the matron would be necessarily detained for a little time in the theatre.

It was nearly half an hour later when Miss Davies came hurrying in full of apologies for the delay which had been the result of an accident to a harvester.

"I suppose agricultural and mining accidents add materially to the number of your patients," I inquired.

"In the autumn there are many harvest injuries, but we do not often get mining accidents," the matron rejoined. "You see in localities where there are many mines there is frequently a miners' hospital, so that injuries may be treated on the spot. We receive patients, however, with letters, from all parts of Cornwall."

"Is this your first experience of the West of England?"

"I have been ward sister and night superintendent at the Royal Infirmary, Bristol, and finally home sister at the Nurses' Home attached. But I have been up and down England a good deal. I was trained at the Royal Hospital, Portsmouth, and then went as staff nurse to the Children's Hospital, Moor Edge, Newcastle-on-Tyne. I was charge nurse at the North Devon Infirmary, Barnstaple, staff nurse at the Poplar Hospital for Accidents, London, and sister at the Cumberland Infirmary, Carlisle, and head sister of the County Hospital, Huntingdon, where I took the matron's holiday duty."

### The Patients.

"How many beds have you here?"

"Only 52. There are two women's wards, with ten beds in each, and one small ward for paying patients There is also an ophthalmic ward with three ophthalmic beds. The eye work is a speciality here. There is the same accommodation for men. We have no wards set apart for children; those under six go with the women, boys over that age are placed with the men. There is also a separate accident ward, but it is very seldom used, and I am glad to say that the isolation block is not wanted often. If we have to get in an extra nurse to assist when we have any particularly arduous cases, I find the nurses' bedroom in the isolation block very convenient to accommodate her."

### The Training of the Nurses.

"What is your regular staff?"

"We have two sisters, and one senior nurse who has been on night duty for 25 years—surely a record work! She has charge of the women's wards, and supervises the work of the probationers in the men's wards. Probationers take night duty for two months at a time. There are five day probationers, and every six months we have a nurse to train for the County Nurses' Association.

"You train for three years?"

"Yes, and lectures are given by the resident medical officers on anatomy and bandaging, etc. The doctor at present in charge—he has been here some time—takes a great interest in the probationers, and does everything he can to help them on. He prefers to take all the classes himself, but I am able to give much general instruction, when I go round the wards every morning with the doctor. The sisters teach in the wards as well."

### Hours and Holidays.

"Do the probationers assist at all in the theatre?"

"Each ward-sister accompanies her own case into the theatre for operation, and takes a probationer with her."

"Are the hours of duty long?"

"They are not short, but a good deal of off-duty time is allowed. The day nurses enter the wards at 7.30 A.M. One evening they leave at 7 o'clock and can then go out until 9 o'clock, or, if they have friends to go to, or on special occasions if they ask, till 10 o'clock. The next day they are off from 2.30 to 5 o'clock. Then upon returning to the wards after tea, which is from 5 to 5.30, they work on till 10 o'clock. They have one day a month off duty, and one calendar month holiday every year. This applies to all members of the staff."

### Uniforms and Salaries.

"Have you any difficulty in obtaining probationers?"

"None whatever. As a rule, I get about eight or nine applications every month, and already the December vacancy is promised. Many of the applicants are the daughters of clergymen or Nonconformist ministers."

"I suppose you provide uniform?"

"Except for the first month, pink and white striped cotton dresses, aprons with square bibs and straps, and Sister Dora caps are given for the use of the nurses by the committee. They also provide a green material frock for outdoor wear, paying for the making, etc., but nurses have to buy themselves a green cloak and a bonnet with veil to match. These they are bound to wear on all occasions except on days off and at holiday time."

"And the salaries?"

"The first year of training the probationers receive £9, the second £14, and the third £15. Sisters receive £25 the first year, and it has been decided to offer £30 for the second year, as an inducement to longer service."

### The Wards.

At this point the matron asked me if I would like to go round the wards. On the way we passed the nurses' sitting-room, which is on the ground floor, and which the Committee, at the matron's suggestion, are having re-papered and made more cheerful. The arrangements upstairs are somewhat unusual. Between each large ward is a smaller ward, containing a stove where special diet can be prepared, where all the medicines, basins, etc., are kept, and where the space next to the fire is screened off for the nurses into a "cosy corner," where they have their tea, or sit down when not actually on duty. Here all the food is prepared for the patients after being brought up from the kitchen. Leading off is a tiny ward kitchen where all the washing up is done, and where the crockery for breakfast and tea is kept.

An Experiment.

Between the wards is a small bedroom for a single paying patient. The experiment of taking paying patients has only been in operation since July, but the matron thinks that it seems likely to prove successful. The charge per week is only 30s., which includes all nursing and ordinary food, but special diet and stimulants are charged extra, and patients are attended by their own doctor. The first and second floors of the hospital are exactly identical, and an equally comfortable little room is available for the use of one male paying patient on the second floor as for the female paying patient on the first floor.

A Garden for the Nurses.

As we looked out of the windows the matron remarked that the Infirmary is particularly rich in garden accommodation. Besides a large kitchen garden which helps to supply the needs of the hospital, there is a garden for the use of the men, another for the female patients, and yet a third for the nurses. The last is shady and pretty, but unfortunately is not available either for tennis or croquet, because the ground is so hilly and it costs so much to level it. Traversing the passages, the matron drew my attention to the arrangements for the prevention of fire, which she said were better than in any infirmary she had ever seen of the same size. Many pails of water stood about, long lengths of hose and hammers for breaking down doors, etc., were suspended from the walls on each landing, and fire practices are to be held every month. Electric communication with the police has also just been completed.

The Chapel and the Theatre.

With justifiable pride my cicerone pointed out the little chapel, formed out of half of the board-room. As meetings are only held there once a year—the receiving-room sufficing for the weekly gathering—the space is well utilised. There is a small altar with a painted glass window at the back and flowers in brass vases on either side of the cross, a brass lectern, altar rails, and a goodly number of chairs. Here, when the curtains are drawn across, the matron reads prayers every day at half-past five, as many as possible of patients, nurses, and domestic staff being present. The chaplain holds service every Sunday afternoon.

"Would you like to see the theatre?" the matron inquired. "I am afraid it will be untidy, because it has been so recently used; but if you will excuse that, you will find it very up-to-date."

As she opened the door and passed in, to her evident satisfaction, we found everything spick and span; the sister had used despatch with admirable effect. The matron informed me that she has charge of the ligatures and instruments, whilst one of the sisters looks after the swabs. As I left the hospital I took a hurried glance at the bright and spacious cubicles occupied by the day nurses and at one of the sisters' bedrooms. The night nurses' quarters are of course in a quiet corner. There was a time when they were under the nurses' sitting-room, but that, happily, is a state of affairs which had ceased long before the advent of the new matron.

### Nurses and Dispensing.

### LONDON SCHOOLS OF PHARMACY.

(*Concluded from page* 302.)

### THE LONDON COLLEGE OF PHARMACY, CHEMISTRY, etc, FOR LADIES.

This is the only college of the kind where none but ladies are taught. It was founded in 1892 by a medical man, who is still at its head, and who is responsible for the lectures. A qualified lady Principal supervises the practical work. The college consists of a private house at 5 Westbourne Park Road, which has been transformed into a series of chemical and pharmaceutical laboratories and class-rooms, and there is a museum containing the pharmaceutical preparations, drugs, chemicals, etc., required for recognition. The largest laboratory is used as a lecture room. Each student has her bench and locker, and here, from ten to half-past four every day but Saturday, she compounds prescriptions and other practical work in the intervals of two lectures daily, each lasting three-quarters of an hour. Evening classes are held by arrangement. Before commencing practical work in the laboratories students are required to provide themselves with the following:—An apron, a duster, "Materia Medica" by Whitla, and "Chemistry" by Luff and Page, a note-book and pencil, also a box of scales and weights, at 2s. The last can be bought at the College. The fees, which are payable in advance, are:—Preparation for the Minor Examination, £10 10s.; preparation for the Major, £8 8s.; preparation for the Apothecaries' Hall, six months, £6 6s.; preparation for the Apothecaries Hall, three months, £4 4s.; preparation for the Apothecaries' Hall, until qualified, £8 8s. Dispensing or any separate subject, three months, £3 3s. The examinations prepared for are the Assistants' examination and the Minor and Major. Some of the students are nurses, and some the daughters of medical men, while a few go through the training for its own sake or as an occupation.

The Principal states that of the failures in examination the largest number are due to want of familiarity with the prescriptions, some of which are not those likely to be met with in ordinary dispensing. He therefore pays particular attention to this subject, and each student makes up ten prescriptions a day.

As a preliminary to joining the college students may take a correspondence course, and the fee for this (£3 3s.) is included when practical work is begun. Fees are payable in advance and are not returnable; but if the work is interrupted by illness or any other unavoidable cause it may be resumed at any time in view of the next examination.

There is an Employment Agency for qualified lady dispensers, and lists of vacancies are sent out as they occur.

Medical book-keeping may be learnt for a fee of one guinea, and a certificate is given.

Resident students are received at 31 Westbourne Gardens, W.

### THE SOUTH LONDON SCHOOL OF PHARMACY.

This school, founded by Dr. John Muter, M.A., F.R.S.Ed., F.I.C., F.C.S. (the well-known public analyst) and carried on by him for more than thirty years, has recently been revived by Messrs. Frank Armstrong, W. F. Mawer, J. Thomas, and A. H. Mitchell Muter, son of Dr. John Muter, whose years of experience as teachers of pharmacy range from twenty to four years. Mr. F. Armstrong, who has had

### NURSES AND DISPENSING—*Continued.*

long experience, is responsible for the teaching of dispensing, pharmacy, and chemistry; Mr. W. F. Mawer, Ph.C., F.C.S., London University, takes botany, materia medica, and Latin; Mr. J. Thomas, B.Sc. London (first-class honours), teaches his favourite subjects, chemistry (practical and theoretical) and physics; and it is largely owing to his efforts that the success obtained in the Major examination by students of this school is due. Mr. A. H. M. Muter, F.I.C., F.C.S., whose strong point is analytical chemistry, has been teaching under his father for some four years. Students wishing to take a post-graduate course in analysis of food and drugs are placed under his care.

Although Dr. John Muter has retired from the more active work of teaching, which for more than 33 years he made his special study, and no longer takes daily classes, he has consented to act as visiting examiner, that is to say, he makes frequent visits to the school, takes one of the classes, and reports on progress. These are surprise visits both to pupil and teacher, and that "a man who can successfully pass the doctor has little to fear from the examinations at the Square" is the opinion of all connected with the school.

The school is at 325 and 409 Kennington Road, S.E. The chemical laboratory was built to Dr. Muter's design, and is so arranged that every student can see the demonstrator and can be seen by him; two rows of benches are sunk below the floor level, and two are above. Each student has gas and water at his elbow and his own set of reagents. The other rooms are a museum and small class-room, a pharmaceutical laboratory fitted with steam plant for the manufacture of galenicals; a histological laboratory, where there is a cabinet containing about 1,000 slides; a large lecture hall, where, if necessary, 200 students can be seated; a laboratory for organic work and gas analysis, and research laboratories. The following are inclusive fees:—For the minor, one term, £10 10s.; two terms, £19 19s.; three terms, £27 10s. For the major, one term, £9 9s.; two terms, £17 17s.; three terms, £25. Students going straight on for the major after passing the minor from this school are allowed 10 per cent. off the major fees. The fee for preparation for the assistants' examinatien of the Apothecaries' Hall, per term, is £5 5s. There is a short term for the October minor examination, for which the fee is £5 5s. Special terms may be had on application for part-time students, also for those who require coaching in chemistry, botany, pharmacy, materia medica, or microscopy for the medical or kindred examinations.

The attention of the school is chiefly devoted to preparation for the examinations of the Pharmaceutical Society, but students wishing to prepare for the Assistants' examination of Apothecaries' Hall can do so; and as their instruction is identical with that necessary for the Minor examination it is bound to be thorough. A few ladies are received.

On entering the school each student receives a set of concise lecture notes, which contain the subject matter of all the lectures. The materia medica notes also serve as a catalogue of the specimens in the museum. Importance is attached to the arrangement of the classes, which are worked so that the attention of the student is not unduly strained by being kept to one subject for a great length of time. The work is changed about every hour.

A special feature of this school is the open entrance scholarship, founded in honour of Dr. John Muter on his retirement from the teaching staff. It is primarily intended as an encouragement to apprentices and young assistants to make good use of spare time. The necessary knowledge may be acquired by a private student, and the value is £27 10s., covering the fee for one session of three terms. If the holder of the scholarship passes the Minor before he July examination (that is, before the end of his year's instruction) he is entitled and expected to remain and attempt the Major during his tenure of the scholarship It is open to all apprentices and assistants who have not received instruction from any school of pharmacy, who have never sat for the Minor examination, and who are at least 21 years of age. Women are eligible provided that they can comply with the conditions. A certain amount of home work is set for students to do in the evenings.

### OTHER LONDON SCHOOLS.

Students may be prepared for the usual examinations by Dr. A. B. Griffiths, F.R.S.Edin., F.C.S., and his assistants, at the Brixton School of Chemistry and Pharmacy, 171 Brixton Road, S.W. Particulars may be had from Dr. Griffiths.

There is a Correspondence Class at Skerry's "Civil Service and University College," 27 Chancery Lane, but the City School of Chemistry and Pharmacy at 33 Chancery Lane has been closed since the issue of the 1902-1903 prospectus.

### BIRMINGHAM.

Pupils are received to learn dispensing at the out-patient department of the Birmingham and Midland Hospital for Women, Upper Priory. Instruction is given by Miss Blanche E. Thompson, who is an assistant of the Apothecaries' Society, a student-associate of the Pharmaceutical Society, and who has been for 20 years dispenser at the hospital. Each pupil must be approved by the committee, and preference is given to those who have passed either the preliminary examination of the Pharmaceutical Society or the College of Preceptors. The usual course of tuition is for 12 to 18 months, practical and theoretical, and it comprises elementary chemistry, materia medica, and Latin, as well as dispensing. The fee for 12 months is £12 12s., for 18 months £15 15s., and though in the case of nurses Miss Thompson arranges for a shorter period if desired, and has got her pupils through in six months, she does not consider it advisable, as it is then simply "cram" work. The student who takes the longer period is much more sure of success, and many of the pupils she has taught have, upon leaving, obtained appointments with doctors and institutions, working for another 18 months for the Minor Pharmaceutical examination, thus making the required three years. They are in this way enabled to hold positions—as many do—as qualified chemists and head dispensers. The hours of attendance are each afternoon, except Saturdays and Sundays, from 1.30 for a variable period. The class consists of six lady students, and this is the 27th year since pupils were first received.

### TRAVEL NOTES AND QUERIES.

ACCOMMODATION IN ALGIERS (E. M.).—I fear your terms are rather low for Algiers; it is an expensive place. The cheapest places I know are the Pension Victoria and Pension Hollandais, both in Mustapha Supérieure, where they offer terms from 7 and 8 francs. Can you speak French fluently? If so, matters are a little easier, because there are a few cheap pensions patronised by the French chiefly that might come within your means, but I do not know them personally. Besides the two houses named above, there are two perfectly respectable hotels—Paris and Genève—where their charges begin at 8 francs, and the Anglo-Suisse, with similar prices. In your place I should go to one of them and look about. At the English library, if you took out a small subscription, you could make inquiries. The addresses of pensions, not of the first class, and therefore naturally anxious to have their names known, are generally to be heard of in English shops and libraries on the Continent, etc. . . . If your stay is to be long, it would be worth while to write to these addresses (English will do) and ask them what arrangements they would make with you.

### Everybody's Opinion.

[Correspondence on all subjects is invited, but we cannot in any way be responsible for the opinions expressed by our correspondents. No communication can be entertained if the name and address of the correspondent are not given as a guarantee of good faith, but not necessarily for publication. All correspondents should write on one side of the paper only.]

### NURSING IN ROME.

"A READER" writes from the Hotel Miramonti, Cortina d'Ampezzo, Tyrol, Austria: Having seen your comments on the "Anglo-American Nursing Home" in Rome, I think it only fair to let you know that English or American visitors to Rome need not depend solely on that institution for a supply of nurses, as highly-trained and fully-qualified American nurses can always be obtained at St. Paul's Home for Nurses (not Nursing Home) at 25 Via Vicenza.

### THE NURSING STAFF AT NEWTON ABBOT.

THE clerk to the Newton Abbot Union writes: With reference to the paragraph in your issue of September 5th as to the nursing staff at this workhouse, I think it only right that I should inform you that the proposal that four (not three) candidates for the appointment of nurse should be asked to appear before the board was carried, and not, as stated in your issue, rejected in favour of the amendment that two nurses should be appointed at that meeting. The guardians of this union invariably interview the candidates before appointment.

[We regret that the report on which our remarks were founded was not correct, and congratulate the Newton Abbot guardians on their adherence to the plan of interviewing candidates before appointment.—EDITOR, THE HOSPITAL.]

### HOME FOR IMBECILE CHILDREN.

"THE SECRETARY OF THE NATIONAL ASSOCIATION FOR PROMOTING THE WELFARE OF THE FEEBLE-MINDED" writes from 53 Victoria Street, London, S.W.: Someone has kindly furnished me with a copy of your interesting paper in which there is marked the answer to query 197 concerning a home for a child subject to fits and at times imbecile. I may point out to you that we should find most if not all of our homes refusing to take children with fits (most often epileptic), though of course it is possible that a very occasional exception might be made. So many epileptics come here hoping to hear of a likely institution to meet their needs that I feel you will like to know this fact to prevent yet more coming, only to be met with disheartening refusal. I have several times seen a kindly reference to our efforts in your pages, and may take this opportunity to send our grateful thanks for your notices.

### Appointments.

[No charge is made for announcements under this head, and we are always glad to receive, and publish, appointments. The information to insure accuracy should be sent from the nurses themselves, and we cannot undertake to correct official announcements which may happen to be inaccurate. It is essential that in all cases the school of training should be given.]

BECKETT HOSPITAL, BARNSLEY.—Miss M. M. Dobbin has been appointed sister. She was trained at the Royal Victoria Hospital, Belfast, where she has since been sister.

FAIR ISLAND, SHETLAND.—Miss Payne has been appointed Queen's Nurse. She was trained for three years at the Portsmouth Infirmary, has been a pupil at the Glasgow Maternity Hospital, and received six months' district training in Edinburgh. She holds the L.O.S. certificate.

FULHAM INFIRMARY, HAMMERSMITH.—Miss S. Margaret Bailes and Miss Hannah Gullick have been appointed sisters. Miss Bailes was trained at Sunderland Union Infirmary, and has since been private nurse at Newcastle-on-Tyne and charge nurse at the North-Western Fever Hospital, Hampstead. Miss Gullick was trained at St. Pancras Infirmary, and has since been charge nurse at Aston Union Infirmary, Birmingham, and private nurse at Northampton.

GREAT SNORING WORKHOUSE, NORFOLK.—Miss Mary Skinner has been appointed superintendent nurse. She was trained at the Cancer Hospital, Brompton, and at Scarborough, and has since been charge nurse at Hemel Hempstead Union.

LISKEARD COTTAGE HOSPITAL.—Miss Alice Brierley has been appointed nurse matron. She was trained at Guy's Hospital, London, and has since been matron at Tetbury Cottage Hospital and matron at the West Highland Cottage Hospital, Oban.

RHONDDA FEVER HOSPITAL.—Miss Rose E. Smith has been appointed matron. She was trained at Worthing Infirmary, and Grafton Street Fever Hospital, Liverpool. She has since been matron of Belvidere Isolation Hospital, Kent.

ROYAL ORTHOPÆDIC HOSPITAL, BIRMINGHAM.—Miss Margaret Nicoll has been appointed matron. She was trained at St. Thomas's Hospital, London, where she was afterwards staff nurse. She has also been sister at the Hospital for Sick Children, Great Ormond Street, Bloomsbury.

ROYAL VICTORIA HOSPITAL, BELFAST.—Miss Edith Blayney has been appointed ward sister. She was trained at Guy's Hospital, London, and holds the L.O.S. certificate.

RUCHILL HOSPITAL, GLASGOW.—Miss Elizabeth Anna Smith has been appointed sister. She was trained at the Royal Hospital, Donnybrook, Dublin, and at the City of Glasgow Hospital, Belvidere. She has since been charge nurse of the male medical ward at the East Parochial Hospital, Dundee.

SCULCOATES UNION INFIRMARY, HULL.—Miss M. L. Mullin has been appointed sister. She was trained at Fir Vale Union Infirmary, Sheffield, where she has since been staff nurse and temporary sister.

### Presentations.

BIRMINGHAM ORTHOPÆDIC HOSPITAL.—Miss Edith Glanville, who is retiring from the post of matron of the Royal Orthopædic Hospital at Birmingham, in consequence of her marriage, has been presented by the general committee with an address recording their sense of her services during a period of ten years, and a cheque.

CHIRK DISTRICT NURSING ASSOCIATION.—Miss L. M. Booker, who is resigning the post of district nurse, at Chirk, which she has held for three years, has been presented with a framed illuminated address signed by a large number of persons who had been her patients, a photograph, and a dressing case containing silver-mounted articles. There were numerous speeches on the occasion, Lord Trevor, who presided, Lady Trevor, Dr. Lloyd, and several past patients, testifying to the skill and kindness shown by the retiring nurse. Miss Booker has been the recipient of many other presents, including a silver cake dish, a silver sugar basin, cream jug and sifter, a violin in case, and a Shetland shawl.

GLASGOW MATERNITY HOSPITAL.—On Monday, August 31st, the nurses and pupils past and present of Glasgow Maternity Hospital presented Sister McFadyen with a handsome travelling rug (Argyle plaid) and straps, and Nurse Locke with a fitted dressing-case. The night sister, in a few well-chosen words, made the presentation, all the nurses at the close singing "Auld lang syne."

### Echoes from the Outside World.

### The King at Balmoral.

On Saturday afternoon the King paid an unexpected visit to Newstead Abbey, the home of the poet Byron. His Majesty inspected the Newstead relics, and saw the spot where the poet's favourite dog was buried. On Monday morning he left Rufford Abbey for Balmoral, travelling by the East Coast route. At Edinburgh and Aberdeen stations he had an enthusiastic reception, and at Ballater, which he reached a little after six, in beautiful weather, there was a guard of honour consisting of 50 men of the 1st Battalion of Royal Highlanders (Black Watch) under the command of Major Hugh Rose. A large crowd occupied the rest of the square. The Prince of Wales, in Highland dress, was present, and afterwards, in company with the King, inspected the guard. Amid great cheering his Majesty and the Prince of Wales subsequently drove off along the North Deeside road to Balmoral, which was reached about seven. The Balmoral Highlanders, in their picturesque dress, under the command of Mr. John Michie, factor, and many of the tenantry, lined the entrance to the Castle on either side, and gave the King a cordial welcome.

### The Turkish Outrages in Macedonia.

On Monday four Bishops of the Church of England protested against the barbarities perpetrated by the Turkish soldiers engaged in the suppression of the rising in Macedonia, and dilated on the duties and responsibilities of this country with regard to them. On the same day details of frightful atrocities in the villages of Monastir and Adrianople were published in the leading journal, whose special correspondent states that it is daily becoming clearer that the intention of the Turks is not only to destroy the insurgents, but as far as possible to exterminate the Bulgarian element, to strike terror into the hearts of all the Christian subjects of the Sultan alike, and to relieve themselves of any anxiety on the score of revolutions for many years to come. No attempt, it is stated, is made to discriminate between guilty and innocent, between rebels and friends, between Bulgarians and Greeks or Wallacks. One of the most revolting incidents described, and witnessed by a foreign officer, was at a Greek village within sight of Florina and visible from the railway. This village, which is, or was, known as Armensko, had to be passed by a Turkish force on its way to attack one of the insurgent bands. Although the inhabitants were not Bulgarians, but Greeks, the village was surrounded, sacked, and many of the inhabitants burnt alive. In a Bulgarian village called Smilevo, which the Turkish troops and Bashi-Bazouks destroyed, they are said to have captured about 60 women, some of whom afterwards escaped and had terrible stories to tell. Both with regard to these and other serious allegations, the Turkish embassy in London declares that there is "no foundation whatever for them."

### The Victoria Cross for a West Surrey Officer.

The King has signified his intention to confer the decoration of the Victoria Cross on Captain Wallace Duffield Wright, of the Royal West Surrey Regiment, for conspicuous bravery during the Kano-Sokoto Expedition in West Africa. The facts are these. On March 14th last Captain, then Lieutenant Wright, with only one officer and 44 men, took up a position in the path of the advancing enemy, and sustained the determined charges of 1,000 horse and 2,000 foot for two hours. When the enemy, after heavy losses, fell back in good order, Lieutenant Wright continued to follow them up till they were in full retreat. The personal example of this officer, as well as his skilful leadership, contributed largely to the brilliant success of this affair. He in no way infringed his orders by his daring initiative, as, though warned of the possibility of meeting large bodies of the enemy, he had purposely been left a free hand.

### Neglected Children.

At the meeting of the British Association in Southport, on Monday, Mrs. Helen Bosanquet read an interesting paper on "Physical Degeneration and the Poverty Line," in the course of which she sought to refute the theory that one third of the population is too poor to maintain itself in physical efficiency. She admitted, however, that many children never attain their proper development, and are greatly in want of better care and feeding. These, she contended, are mainly the children whose parents have the means to nurture them properly, but are too ignorant, or too careless, to do so. The evil, being not mainly due to poverty, could not be met by subsidising the parents' earnings, nor would school feeding, whether free or paid for, be sufficient to satisfy all the needs of the children. They could only be met ultimately by educating women to a more adequate fulfilment of their duties as wives and mothers, and meanwhile by dealing with neglected children individually.

### The Disastrous Gale.

Enormous destruction was wrought by the gale which swept over England at the end of last week. Buildings were overthrown or partially demolished in many towns on the coast, while thousands of pounds worth of costly sea fronts, esplanades, and works were destroyed. At Dover the damage to the new harbour works is reported to exceed £40,000; Weston-super-Mare has been injured to the extent of £20,000 whilst at Hastings the concert pavilion is wrecked and 50 bathing machines were smashed to bits. Many gallant rescues by the lifeboats are chronicled. At Clay, in Suffolk, a steam yacht became disabled, and the Sheringham lifeboat journeyed a distance of nine miles and was then launched through tremendous seas on Friday morning. She succeeded in reaching the vessel after much difficulty and towed her into safety. The men were working altogether over 20 hours. At New Quay in Cornwall a vessel with five or six hands foundered before the lifeboat could reach her. At St. Ives the lifeboat saved four lives; at Looe it went out twice and saved six lives; at Port Isaac it landed six men from a French brig, and gallant work was done also by the brave rescuers on the North Wales, Dorsetshire, Sussex, Essex, and other coasts. A great number of dead bodies have been washed ashore, and many lives were lost through the floods and falling timber.

### "Richard II." at His Majesty's Theatre.

The revival of "Richard II." at His Majesty's Theatre marks the commencement of the autumn theatrical season. This version of Shakespeare's play is in three acts and eleven scenes, and neither pains nor expense have been spared upon the scenery, which is really magnificent. The play would be worth seeing if only because of the splendid historical show. Of the series of brilliant stage pictures the lists at Coventry, Bolingbroke's entry into London, and the Coronation in Westminster Abbey are the most notable, but all are interesting and successful. Mr. Tree is, of course, King Richard, and among other fine touches the monarch's silent farewell to his few personal friends as he leaves Westminster Hall merits special recognition. Miss Lily Brayton displays unexpected resources in her rendering of the Queen; Mr. Oscar Asche is effective as Bolingbroke; Mr. Brandon Thomas is in his element as the venerable John of Gaunt; Mr. W. Haviland makes an excellent representation of Mowbray of Norfolk; and Mr. Lionel Brough, in the small part of the Gardener, is impressive. The dresses are as tasteful as they are rich, and Miss Brayton's costume in the scene in Windsor Castle, where she and her ladies are singing in the oriel window, with the storm raging outside, is particularly charming.

### A Book and its Story.

NEW WORK BY THE AUTHOR OF "ON THE FACE OF THE WATERS."*

A THIRD edition following quickly on the two first issues of Mrs. Steel's Indian Sketches is sufficient proof of their popularity. Written with the insight and familiarity of one long resident in the country, they cannot fail to be interesting to those who know Anglo-Indian life well. One of the best told, and of the most far-reaching interest is, "Gold, Frankincense, and Myrrh." The scene opens on Christmas Eve, in an Indian cantonment, in the verandah of a house in which the wife of the colonel of an Indian cavalry regiment has remained during his absence in South Africa. "She was one of those women, loveable utterly, not always reasonable, who find solace in dramatising their own sorrows. So when, two years before, her husband had been ordered to Africa on staff duty, she had remained behind in the big house, sharing it with a friend, continuing religiously to care for all the things for which her absent soldier had cared—even for the regiment—which was still so proud of its colonel at the Front."

In the station Boer prisoners had an encampment, and the Colonel's wife had devised a Christmas tree for their amusement. "It seemed to this woman, militant to the heart's core, yet sentimentally pitiful, it had seemed appropriate that Boy—son of the only fighting father in the station—should play the part of the 'Christ Kind,' the bringer of good gifts at the Christmas Tree . . . and Boy's mother thought that her little son, with his aureole of red hair and grave baby face, so like the absent hero, would look sweet in the part." But Boy could not agree with his mother on this point. In reply to her pleadings that to love his enemies was part of the Christmas message, he replied, "I'd wather fight them, like daddy," drawing from its scabbard a tiny sword of strict regimental pattern to support his argument. This treasured toy he had refused to discard for the white robe—a night-gown with little pretence wings sewn on to it—in which he was to appear as the Christ-child.

The Boer general Viljoen was among the prisoners and had escaped. Boy's imagination had been aroused by talks with Tommies in the camp. They had fostered his militant attitude by declaring that he, Viljoen—"Vile John" they called him—would kill all the English children he met because "they killed his."

"But," replied his mother, "they died, dear," and so you must be very sorry for him. Think how sad it should be if ——" The thought produced a sudden caress, a sudden glisten in the grey eyes. "Now, Boy of mine, let me take that thing off." And the child was sent off to rest in charge of the ayah who was to bring him dressed for the gift-giving, at eight o'clock in the evening.

But a storm of rain and wind was gathering, and when the evening came it swept all before it. The carriage had come for the ayah and child to be driven to the Christmas festivities. "Hurry up," called the coachman. "It has begun down the road like the storm of God. Bring the child! It were best he was soon in safety." Bring the child! How, when Boy, with his little pretence wings, his little sword that was not all pretence, was not to be found? Boy, when half asleep, had slipped out into the garden, sword in hand, to play the game of hunting for Viljoen. If he found him, he would, of course, kill his father's enemy. At first, outside in the deepening darkness of the coming storm, he had not been afraid. He was too engrossed in the pursuit of the foe, to notice the change from light, to darkness. But stumbles over the rough road in the belt of jungle adjoining the garden, in his long white robe, became wearisome, and, "when the first flash of lightning turned the wilderness around him into black and white shadows," his courage began to fail, and he ran blindly on, too brave to call out. In its flight, "That quaint little figure, sword in hand, had, almost at first, run tilt against a man who was crouching to leeward of a big tuft of tiger grass—a man whose head was buried in his crossed arms, but who sprang to his feet with a curse at the unmistakable touch of humanity. Then, as a flash of lightning showed him the white robe, the wings, the golden aureole of hair, fell back faltering." The child, from sheer exhaustion, had fallen fast asleep in the arms of the fugitive Viljoen. He lay, "cuddled warmly on a big, broad breast, against a big brown beard." Awakening to find himself in the human shelter which, if alien, was welcome to the little wanderer, Boy, in reply to Viljoen's inquiry, "What dost thou here?" replied, "Oh, please, take me home. I wanted to kill vile John with the sword as Kunder sharped; but now I'd wather, please, give the Chrismus fings—the peace you know, an' all that—please, sir, I weally would wather——" A sudden smile half bitter came into the man's bewildered face. "You wanted to kill vile John," he said, in English. "Why?" "Oh, I don't know—but I don't want to now—I'd wather bring the peace." "God in heaven!" exclaimed the man, reverting to his own tongue. "We shall be drowned if we stop here. Come, little rat! Let us find a spot where we can keep dry." The weird mingling of the storm with the tragic elements of the situation, the pathos of it, too, are brought into strong relief in the succeeding description of the Boer general and the little English child. He carried him with difficulty into a place of shelter and safety, too near the cantonment for him to share it. Before laying his little burden down and departing, he chose a sort of cattle shed, and there he drew a pile of straw into a dry corner and laid Boy down, again asleep, upon it. In the dawn Viljoen was seen guarding the door with a still, stern look on his face. "You will find the child lying in the manger," he said to those who were seeking him; "bring your offerings, I have brought mine." There are other characters and other incidents, but we have only dwelt on the more striking ones. The story reads like a true one, and the words "a little child shall lead them," recur naturally as we put it down.

"In a Fog" is an amusing character sketch of an Irish army medical officer at a remote station who, in charge of fifteen convalescent patients, kept at bay the mutineers in a thick mist. Having summoned his motley corps on the alarm being given of the approach of the enemy, and leaving as "man in charge" a crippled patient with a pair of horse pistols, "by way of consolation," and instructions to hold the fort as long as possible, and prevent the rascals from touching even the drugs, he collected his men together, fourteen all told, without the absent Tompkins, and sallied forth under the cover of the fog. After deciding to arrange the men in flank on an adjacent zigzag, he inquires the distance between each. "Smith, in the fog, thought a moment or two. 'Close on a mile, more or less, Sir, and there's four of them.' 'Say three-quarters, and we are fifteen. No, it's fourteen, for we had to leave poor Tompkins.' 'Beg pardon, Sir,' came a voice from the fog, 'Tompkins present; came on all fours down the short cut, quite easy.' 'Fifteen,' corrected the doctor calmly, 'fifteen into twelve hundred yards . . . See here, we're not fifteen; we're fifteen hundred.' The cripples broke out into a faint cheer. 'We're fifteen hundred strong, an' we're each of us a hundred men, and two officers,' called the doctor. The jest and earnest of it what tongue can tell?"

* "In the Guardianship of God." By Flora Steel. (Heinemanns. 6s.)

### For Reading to the Sick.

"OUR LIGHT AFFLICTION."

Lord ! dost Thou call this our affliction "Light?"
Is all this anguish little in Thy sight?

"Child, bring thy balance out. Put in one scale
All thine afflictions; give them in full tale;
All thy bereavements, grievances, and fears;
Then add the utmost limit of man's years:
Now put My Cross into the other side,
That which I suffered, when I lived and died."

I cannot, Lord; it is beyond my might:
And lo! my sorrows are gone out of sight!

"Then try another way. Put in the scale
The glory now unseen, behind the Veil;
The glory given to be thine own estate;
Use that 'exceeding and eternal weight:'
Which kicks the beam?"

Ah! Lord, Thy word is right,
Thus weighed, my sorrow doth indeed seem "light."

*C. M. Noel.*

In all that befalls ourselves we are not our own, but Christ's, all that we call ours is His; and when He takes it from us—first one loved treasure, then another, till He makes us poor, and naked, and solitary—let us not sorrow that we are stripped of all we love, but rather rejoice for that God accepts us; let us not think that we are left here, as it were, unreasonably alone; but remember that, by our bereavements, we are in part translated to the world unseen. He is calling us away, and sending on our treasures. The great law of Sacrifice is embracing us, and must have its perfect work. Like Him, we must be made "perfect through suffering." Let us pray Him, therefore, to shed abroad in us the mind that was in Christ; that, our will being crucified, we may offer up ourselves to be disposed of as He sees best, whether for joy or sorrow, blessing or chastisement; to be high or low; to be full or suffer need; to have many friends, or to dwell in a lonely home; to be passed by, or called to serve Him and His Kingdom in our own land, or among people of a strange tongue; to be, to go, to do, to suffer as He wills, even as He ordains, even as Christ endured, "Who, through the Eternal Spirit, offered Himself without spot to God."—*H. E. Manning.*

Rejoice that you are made free of the holy brotherhood of mourners, that you may claim your place too, if you will, among the noble army of martyrs. Rejoice that you are counted worthy of a fellowship in the sufferings of the Son of God. Rejoice and trust on, for after sorrow shall come joy. Trust on; for in man's weakness God's strength shall be made perfect. Trust on, for death is the gate of life. Endure on to the end, and possess your souls in patience for a little while, and that perhaps a very little while. Death comes swiftly, and more swiftly still, perhaps, the day of the Lord. The deeper the sorrow, the nearer the salvation.

*Charles Kingsley.*

Teach me to live—my daily cross to bear,
Nor murmur though I bend beneath its load,
Only be with me; let me feel Thee near,
Thy smile sheds gladness on the darkest road.

Teach me to live and find my life in Thee,
Looking from earth and earthly things away.
Let me not falter, but untiringly
Press on, and gain new strength and power each day.

*E. E. Burman.*

### Notes and Queries.

**FOR REGULATIONS SEE PAGE 307.**

*Stewardess.*

(258) I am a trained nurse and am anxious to travel; will you kindly tell if I could get a post as stewardess on board a ship, and what position one would hold as such, and where should I apply for such information?—*M. C. T.*

*Nurses on Liners.*

(259) Can you give me any information as to whether any of the steamship companies have adopted the plan mentioned in an article in your paper, viz., that of having trained nurses on board their vessels?—*M. B.*

Information respecting intended action on the part of any of the steamship companies can only be obtained by writing to them individually; but Miss Penn, The Cottage, New Shoreham. Sussex, might be glad if our correspondents would communicate with her.

*Charity.*

(260) Can you help me to procure £10 for a family who are in great want through no fault of their own?—*Nurse Emily.*

It is impossible to help every case of distress with which a nurse comes in contact.

*Convalescent Homes.*

(261) Can you give me the addresses of any convalescent homes where gentlepeople of limited means are taken for a fee of about one guinea a week?—*R. C. H.*

There is a fairly large choice of convalescent homes at the price you mention. See "Burdett's Hospitals and Charities." A short advertisement would bring you a number of replies.

*Post. Home.*

(262) 1. Will you kindly tell me if it is possible for a nurse to obtain a post as lecturer in any society, or under any Town, or County, Council? 2. Can you kindly give me any idea of the cost of fitting up a private nursing home to accommodate, say, 12 medical and surgical cases?—*Nurse C. B.*

1. See article on "Lecturers" in the "Englishwoman's Year-Book," which can be obtained from the Scientific Press or any bookseller. There are several agencies employing women lecturers, and the County Councils have a large number all over the country. 2. It is quite impossible to say. Any of the firms supplying hospital requirements would give you an estimate on receiving full particulars.

*Hospital Training.*

(263) Can you tell me if I could get into a London hospital, my age is 19?—*Oxonian.*

You are four years too young for general training anywhere.

*Recognised Hospital.*

(264) Will you kindly let me know the number of beds for a recognised hospital?—*Anxious.*

Your question is too indefinite. Do you mean infirmaries recognised by the Local Government Board?

*Midwifery.*

(265) Will you inform me where I could be trained for midwifery free, or at a fee not exceeding five pounds?—*F. E. D.*

There is no free training in midwifery, nor at the fee you name. Consult the Secretary, the Central Midwives Board, 6 Suffolk Street, Pall Mall, S.W., as perhaps some of the new associations now being formed to provide midwives for country districts might give you training in return for a period of service.

*Medical Terms.*

(266) Will you please tell me where I can obtain "Hadden's Pocket Vocabulary of Medical Terms with Pronunciation" which was recently mentioned in The Hospital, and the price?—*L. W.*

Through any bookseller. The Publishers are Hadden, Best and Company, Salisbury Square, E.C. The price is 2s. The Scientific Press will procure all books required by nurses.

### Standard Nursing Manuals.

"The Nursing Profession: How and Where to Train." 2s. net; 2s. 4d. post free.

"Nursing: Its Theory and Practice." (Revised Edition). 3s. 6d. post free.

"Surgical Ward Work and Nursing." (Revised Edition). 3s. 6d. net.; 3s. 10d. post free.

"Practical Guide to Surgical Bandaging and Dressings." By Wm. Johnson Smith, F.R.C.S. 2s. post free.

"Practical Handbook of Midwifery." (New Edition). 6s. net; 6s. 3d. post free.

"Notes on Pharmacy and Dispensing for Nurses." 1s. post free.

"Fevers and Infectious Diseases." 1s. post free.

"The Art of Massage." (New Edition). 6s. post free.

## Figures and Tables

**Figure f1:**
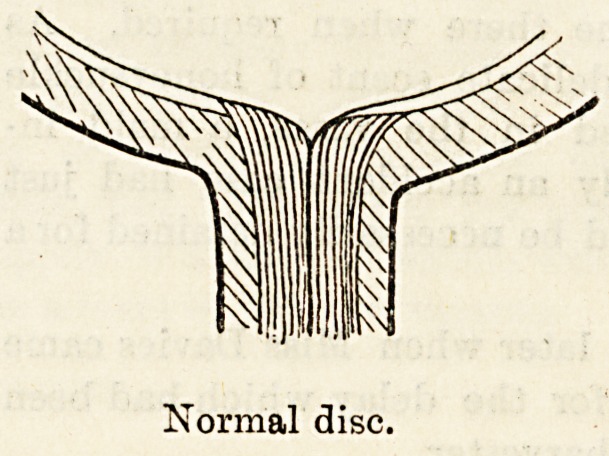


**Figure f2:**